# Inter-plant communication through mycorrhizal networks mediates complex adaptive behaviour in plant communities

**DOI:** 10.1093/aobpla/plv050

**Published:** 2015-05-15

**Authors:** Monika A. Gorzelak, Amanda K. Asay, Brian J. Pickles, Suzanne W. Simard

**Affiliations:** Department of Forest and Conservation Sciences, University of British Columbia, Vancouver, BC, Canada, V6T 1Z4

**Keywords:** Complex adaptive systems, ectomycorrhiza, forests, mycorrhizal networks, plant behaviour, plant communication

## Abstract

Adaptive behaviour of plants, including rapid changes in physiology, gene regulation and defence response, can be altered when linked to neighbouring plants by a mycorrhizal network (MN). Mechanisms underlying the behavioural changes include mycorrhizal fungal colonization by the MN or interplant communication via transfer of nutrients, defence signals or allelochemicals. We focus this review on our new findings in ectomycorrhizal ecosystems, and also review recent advances in arbuscular mycorrhizal systems. We have found that the behavioural changes in ectomycorrhizal plants depend on environmental cues, the identity of the plant neighbour and the characteristics of the MN. The hierarchical integration of this phenomenon with other biological networks at broader scales in forest ecosystems, and the consequences we have observed when it is interrupted, indicate that underground ‘tree talk’ is a foundational process in the complex adaptive nature of forest ecosystems.

## Abstract (precis)

Trees can communicate with each other through networks in soil. Much like social networks or neural networks, the fungal mycelia of mycorrhizas allow signals to be sent between trees in a forest. These mycorrhizal networks are effectively an information highway, with recent studies demonstrating the exchange of nutritional resources, defence signals and allelochemicals. Sensing and responding to networked signals elicits complex behavioural responses in plants. This ability to communicate ('tree talk') is a foundational process in forest ecosystems.

## Introduction

A mycorrhiza is typically a mutualistic symbiosis between a fungus and a plant root, where fungal-foraged soil nutrients are exchanged for plant-derived photosynthate (Smith and Read 2008). The extent of fungal mycelium in the soil is vast and the mutualisms between the fungal species and host plants are usually diffuse, enabling the formation of mycorrhizal networks (MNs). These MNs are composed of continuous fungal mycelia linking two or more plants of the same or different species. The MN can thus integrate multiple plant species and multiple fungal species that interact, provide feedbacks and adapt, which comprise a complex adaptive social network. The MN is considered ecologically and evolutionarily significant because of its positive effects on the fitness of the member plants and fungi. Our understanding of this significance derives from evidence that MNs influence the survival, growth, physiology, health, competitive ability and *behaviour* of the plants and fungi linked in the network. How the MN affects the member plants and fungi is increasingly understood to involve plant–fungal–plant communication, and may involve biochemical signalling (Song *et al.* 2010; Babikova *et al.* 2013), resource transfers (Francis and Read 1984; Simard *et al.* 1997*a*, *b*; He *et al.* 2009; Teste *et al.* 2009) or action-potential-driven electrical signals (Baluŝka *et al.* 2006; Kai *et al.* 2009). The responses of the plants and fungi to this communication are rapid, and thus can be described as behavioural responses, allowing us to refocus our understanding of the significance of MNs through the lens of plant behaviour. Here, we provide insight into the mechanisms and conditions through which MNs may influence plant behaviour, and discuss the potential circumstances and consequences for the ecology of plant communities and the evolution of plant–fungal interactions. We finish with a discussion of how MN influences on plant behaviour are congruent with viewing plant communities as complex adaptive systems. Gaps in our understanding of these patterns and processes are highlighted.

## Mycorrhizas

Mycorrhizal associations of plants have large-scale ecosystem-wide consequences (Averill *et al.* 2014; Dickie *et al.* 2014). The magnitude of their importance is likely due to the proclivity of most terrestrial plants to form them (Smith and Read 2008; Brundrett 2009). The origins of this symbiosis are thought to be ancient and have been proposed as a mechanism for facilitating land colonization by plants 400 Mya (Redecker *et al.* 2000; Humphreys *et al.* 2010). The mycorrhizal symbiosis is a many-to-many relationship: plants tend to form symbioses with a diverse array of fungal species (broad host receptivity) and likewise, fungal species tend to be able to colonize plants of different species (broad host range). While most mycorrhizal fungi are broad host generalists, forming diffuse mutualisms, a few appear to be specialists, occurring exclusively on a single host (Lang *et al.* 2011).

Plant species tend to display fidelity to specific classes of mycorrhizal fungi, and entire ecosystems are often dominated by one class or the other. By far the most ubiquitous class is arbuscular mycorrhizal fungi (AMF), obligate biotrophs in the Glomeromycota that form arbuscules and sometimes vesicles within the root cells of hosts (Smith and Read 2008). Arbuscular mycorrhizal fungal symbioses dominate temperate grasslands, tropical forests and agricultural systems, but also associate with some temperate forest trees, such as members of the Cupressaceae and Aceraceae families (Brundrett 2009). The AMF are microscopic with few morphological features to distinguish between species, and are classified by spore appearance and molecular markers (Rosendahl 2008), and continue to stir controversy due to the atypical genetics of these fungi (Koch *et al.* 2004).

The other major class of mycorrhizas is the ectomycorrizal fungal (EMF) class. Although fewer plant species have been found to form symbioses with EMF, in comparison to AMF (Brundrett 2009), the hosts of EMF tend to be widely dispersed, abundant and dominant members of their assemblages. For example, EMF hosts include most coniferous trees (all of the Pinaceae), the majority of woody shrub species in temperate and boreal forests and the Dipterocarpaceae, which results in EMF also being common in tropical forests. Root tips harbouring EMF are distinguishable by macroscopic features (Peterson *et al.* 2004) including: (i) the mantle (a fungal sheath that encases a colonized root tip) and (ii) extramatrical mycelium (diffuse hyphae that extend out into the surrounding soil). Some species of EMF form epigeous mushrooms and others form hypogeous truffles, predominantly from the phyla Basidiomycota and Ascomycota. Ectomycorrizal fungi appear to have evolved separately in multiple plant families, with as many as 66 incidences identified thus far from phylogenetic evidence (Tedersoo *et al.* 2010). Some exceptional plant families and genera are capable of forming viable symbioses with EMF and AMF simultaneously (e.g. Salicaceae, *Eucalyptus*). Whether an ecosystem is dominated by AMF or EMF associations has large-scale consequences for resource availability, as AMF systems have been recently demonstrated to have lower soil C : N ratios than those dominated by EMF, indicating fundamentally different nutrient cycling regimes, resulting in more carbon sequestered in EMF forests (Averill *et al.* 2014).

Both EMF and AMF fungi have been demonstrated to form networks. Their structure and known associations are noted here to highlight the differences between them, and to point out that despite these differences, they both appear to be able to affect plant behaviour changes through the formation of networks. We do not fully explore the potential mechanisms by which communication is occurring through mycorrhizas, as this topic is reviewed elsewhere (Barto *et al.* 2012); rather, we synthesize the evidence for and consider the potential extent and ecosystem consequences of this phenomenon.

## Mycorrhizal Networks: Structure and Function

**Figure 1.**
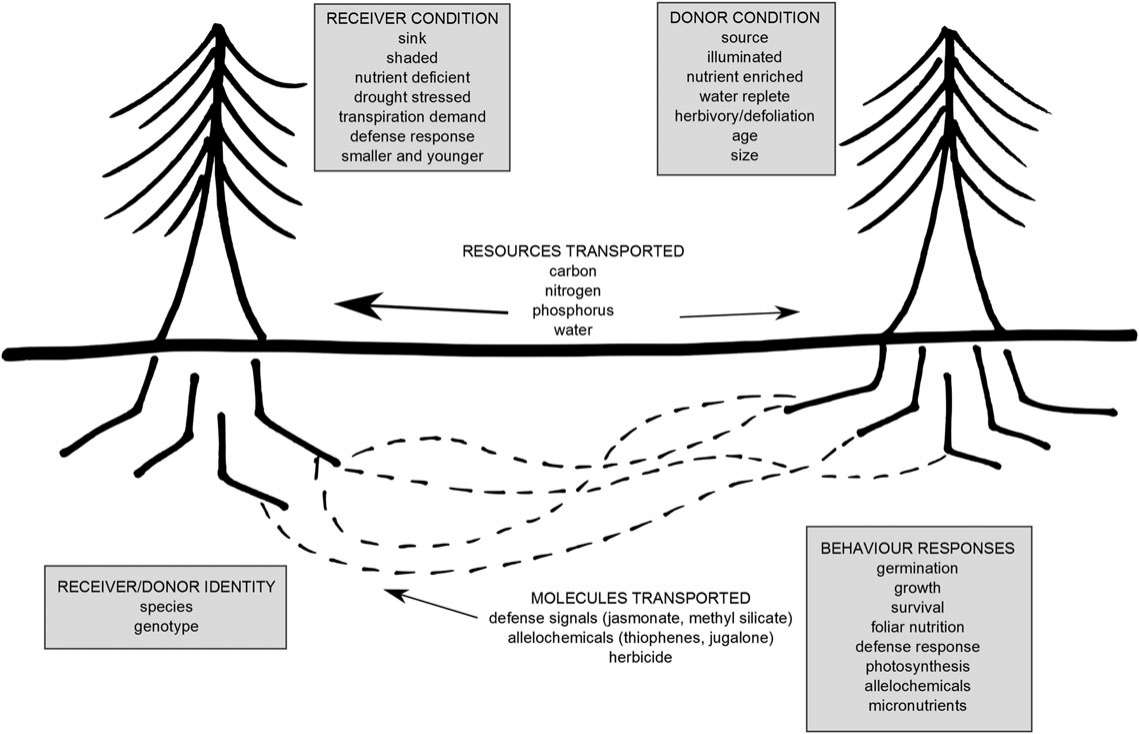
Schematic of resources and signals documented to travel through an MN, as well as some of the stimuli that elicit transfer of these molecules in donor and receiver plants.

An MN is formed when multiple plants are linked belowground by a continuous AMF (Kiers *et al.* 2011) or EMF mycelium (van der Heijden and Horton 2009; Beiler *et al.* 2010) (Fig. 1). Networks can be exclusive to a subset of plants able to form mycorrhizas with the same fungi, potentially linking members of a single species [for example Beiler *et al.* (2010)]. The MN can also be inclusive, as may be the case for AMF systems, where linkages can occur between multiple plant and fungal species (Molina *et al.* 1992). Evidence for the occurrence of MNs has been accumulating for half a century (Björkman 1970; Reid and Woods 1969; Francis and Read 1984; Newman 1988), but their significance for ecosystems has been pursued intensively only in the past two decades (Simard *et al.* 1997*b*; Fitter *et al*. 1998). We now know that MNs can influence plant establishment (Dickie *et al.* 2002; Nara 2006), survival (Horton and Bruns 2001; Teste *et al.* 2009; Bingham and Simard 2011), physiology (Wu *et al.* 2001, 2002), growth (Teste *et al.* 2010) and defence chemistry (Song *et al.* 2010, 2014; Babikova *et al.* 2013). This influence is thought to occur because the MN serves either as a pathway for interplant exchange of resources and stress molecules or as a source of fungal inoculum (Fig. 1) (see reviews by Simard *et al.* 2012, 2013, 2015). For instance, anastomosis with existing MNs of established plants is considered the most common mechanism for mycorrhizal fungal colonization of regenerating plants *in situ* (van der Heijden and Horton 2009; Kariman *et al.* 2012). Colonization of establishing seedlings by MNs enables them to acquire sufficient soil nutrients for root and shoot growth and hence survival (Teste *et al*. 2009; Kariman *et al.* 2012).

As with mycorrhizal colonization, interplant resource and signal fluxes through MNs have the potential to alter plant behaviour. These fluxes have been shown to include carbon (Simard *et al.* 1997*a*, *b*), water (Egerton-Warburton *et al.* 2007; Bingham and Simard 2011), nitrogen (Teste *et al.* 2009), phosphorus (Eason *et al.* 1991), micronutrients (Asay 2013), stress chemicals (Song *et al.* 2010, 2014; Babikova *et al.* 2013) and allelochemicals (Barto *et al.* 2011), and can occur between plants of the same or different species. Understanding the potential effects of these fluxes on plant behaviour, however, first requires an understanding of transfer processes, and the factors that regulate these processes. For instance, interplant resource exchanges are thought to be regulated by source–sink relationships within the MN, where one plant that is rich in nutrients serves as a source (donor) of compounds for a neighbouring plant that is poor in nutrients, which thus serves as a sink (receiver) (Simard *et al.* 2012). The long-distance transport of carbon and/or nutrients appears to occur predominantly by advective mass flow driven by the source–sink gradient generated by these interplant nutrient differences (Simard *et al.* 2012). Mass flow can also be generated by fungal mycelium growth, and diffusion or active transport mechanisms may operate during active fungal cell expansion at growing mycelium fronts (Heaton *et al.* 2012).

The primary importance of plant–sink strength in governing the magnitude and direction of resource transfer through MNs is illustrated in studies showing transfer of carbon to rapidly growing EMF saplings with high transpiration rates, or to shaded EMF seedlings with high respiration demands for survival and growth (Read and Bajwa 1985; Simard *et al.* 1997*a*; Teste *et al*. 2009, 2010). Organic compounds are thought to enter the transpirational stream of the receiver plant via the xylem, and then be actively transported to rapidly expanding biosynthetic tissues. Plant source strength also drives transfer under certain conditions. This was demonstrated in the rapid transfer of labile carbon from the roots of injured EMF seedlings to healthy neighbours (Song *et al.* 2014), and in the transfer of nitrogen from N_2_-fixing or fertilized source plants to non-N_2_-fixing sink plants (He *et al.* 2009). Increasing source strength by CO_2_ fertilization of plants in AMF networks, in contrast, has had no effect on carbon transfer (Fitter *et al.* 1998). Although either source or sink strength may dominate under certain circumstances, it is more likely that the simultaneous behaviour of both source and sink plants (and sources and sinks within the mycelium itself) influences carbon and nutrient transfer through MNs. For instance, the direction and magnitude of carbon transfer changed over a growing season due to simultaneous changes in phenology, and hence source and sink strength, of different plants involved in an EMF network in mixed temperate forest (Philip 2006) and Low Arctic Tundra (Deslippe and Simard 2011). Carbon and nitrogen are thought to travel through MNs together in simple amino acids (Simard *et al.* 2015). These molecules travel through the MN rapidly, moving from donor plants to the fungal mycelium within 1 or 2 days (Wu *et al.* 2002; Heaton *et al.* 2012) and to the shoots of neighbouring plants within 3 days (Wu *et al.* 2002).

Using experimental designs that prevent the aboveground transfer of volatile organic compounds (VOCs), as well as controlling for the formation of an MN, stress signals have been shown to transfer from injured to healthy plants through MNs even more rapidly than carbon, nutrients or water. Herbivore- and pathogen-induced stress responses were up-regulated in undamaged neighbours in as little as 6 h following insect or fungal infestation of donor plants linked in AMF MNs (Song *et al.* 2010, 2014; Babikova *et al.* 2013). Song *et al.* (2014) found up-regulation of four defence-related genes in healthy neighbours 6 h after AMF tomato donors were infested with the leaf-chewing caterpillar *Spodoptera litura* Fabricius, likely in response to signalling via the jasmonate pathway through the MN. They showed that production of defence enzymes increased receiver resistance to pests, as indicated by lower weight gain and hence lower fitness of the herbivore. In an earlier study, Song *et al.* (2010) infested AMF tomato plants with the foliar necrotrophic fungus, *Alternaria solani*, and similarly found that six defence genes encoding for defence enzymes were activated after 65 h in the un-infested neighbours. In this study, the use of mutant controls and the genes that were up-regulated suggested that salicylic acid and jasmonic acid pathways were involved in signalling (or signal detection ‘eves-dropping’) through the MN. In an MN linking EMF Douglas-fir and ponderosa pine, defoliation of Douglas-fir with the insect *Choristoneura occidentalis* (western spruce budworm) induced defence enzyme production in neighbouring linked ponderosa pine within 24 h of infestation (Song *et al.* 2015). These studies clearly show a rapid physiological response of recipient plants to stress signalling transmitted through MNs, even in plants of a different genus, indicating a shift in plant behaviour to protect fitness.

## Plant Behaviour

Plant behaviour is defined as a change in plant morphology or physiology in response to environmental stimuli, including responses to chemicals, light and water, that occurs within a plant's lifetime (Karban 2008). Phenotypic plasticity, the ability of an individual to alter its traits in response to the environment, is a defining feature of plants. McNickle *et al.* (2009) define behaviour as the expression of plant plasticity that is like a decision point, where each choice involves trade-offs that will affect fitness. The most studied plant behaviours involve alteration of aboveground morphology to optimize access to sunlight (Smith 2000; Novoplansky 2009), or of reproductive or defensive traits to deal with environmental heterogeneity (Karban 2008). Plant behaviour responses can also occur belowground, in foraging for nutrients and water (Hodge 2004) and in response to root competition (Cahill *et al.* 2010). Since all forest plants form obligate symbioses with mycorrhizal fungi, plant behavioural responses must also include any physiological changes due to their mycorrhizal status (e.g. changes in fine root morphology and/or production following fungal colonization). The relative speed of the change, the presence of stimuli and non-permanence of the change (e.g. short-term production of defence chemicals) distinguish plant *behaviour* from other chemically mediated processes (Karban 2008).

## Mycorrhizal Network Effects on Plant Behaviour

Plants that are connected via an MN can rapidly modify their behaviour in response to fungal colonization and interplant biochemical communication. Plant behavioural responses that have been measured thus far include rapid changes in mycorrhizal colonization, root growth, shoot growth, photosynthetic rate, foliar nutrition, foliar defence chemistry and defence response (Fig. 1, Table 1). For instance, plant hosts have responded to mycorrhizal colonization via MNs by adjusting production of fine roots (e.g. Pickles *et al.* 2010), probably due to the production of plant auxins, and this has been noted in both EMF (Frankenberger and Poth 1987; Gay *et al.* 1994; Karabaghli-Degron *et al.* 1998; Scagel and Linderman 1998; Felten *et al.* 2010) and AMF systems (Hanlon and Coenen 2011). These varied types of plant behavioural responses have occurred in cases where the neighbouring plants linked in the MN (i) are of the same or different species and (ii) are kin (of the same parental lineage) or strangers. These observations raise questions about the evolutionary consequences of MN-related interactions. Plant behavioural changes have qualities and quantities that can substantially alter the community ecology of a site, including intra- and interspecific interactions, species co-existence and biodiversity. Changes to the fitness of connected neighbouring plants and associated organisms can also have ecosystem-wide impacts. In this section, we discuss evidence that MNs can influence plant behaviour, with ripple effects on plant community and ecosystems ecology.

**Table 1.** List of recent studies documenting plant behaviour changes mediated through connection to MNs.

Plant behaviour mediated through MNs	Plant host	MN forming fungus	References
Increased foliar N	*Ambrosia artemisiifolia* L.	*Glomus intraradices*	File *et al*. (2012)
Increased mycorrhization of kin through MN			
Foliar defence chemistry	*Vicia faba*	*Glomus intraradices*	Babikova *et al.* (2013)
Defence response to pathogenic fungus	*Lycopersicon esculentem* Mill.	*Glomus mosseae*	Song *et al.* (2010)
Defence response to leaf-chewing caterpillar	*Lycopersicon esculentem* Mill.	*Funneliformis mosseae*	Song *et al*. (2014)
Increased survival, growth and carbon transfer	*Pseudotsuga menziesii* var. *glauca*	Natural EMF	Teste *et al.* (2009)
Improved survival under drought stress	*Pseudotsuga menziesii* var. *glauca*	Natural EMF	Bingham and Simard (2011)
Improved survival with established MN	*Pseudotsuga menziesii* var. *glauca*	Natural EMF	Bingham and Simard (2012)
Growth after disturbance, carbon transfer	*Pseudotsuga menziesii* var. *glauca*	*Rhizopogon vinicolor*	Teste *et al*. (2010)
Increased photosynthesis, carbon transfer	*Pseudotsuga menziesii* var. *glauca*	Natural EMF	Simard *et al.* (1997*a*)
	*Betula papyrifera* Marsh.		

The impacts of MN-mediated resource transfer on plant behaviour are evident in the regeneration success of mono-specific and mixed forests. In the interior Douglas-fir forests of western North America, the transfer of carbon, nitrogen and water from older trees to regenerating seedlings through an MN has been associated with rapid increases in net photosynthetic rates (Teste *et al.* 2010), shoot water relations (Schoonmaker *et al.* 2007) and shoot and root growth of the young seedlings (Teste *et al.* 2009). These responses were linked to improved seedling survival and productivity, and hence regenerative capacity of the forest. Whether they were caused by the observed carbon, nitrogen or water transfer, or by some other facilitative effect of the MN, remains unknown. However, similar shifts in Douglas-fir seedling behaviour in response to MNs have been repeatedly observed in a range of regional climates (Simard *et al.* 2012). In a separate study, water transfer from replete to drought-stressed conspecifics through MNs was also associated with increased growth (Bingham and Simard 2011). Here, the receiver seedlings accessed water through the MN, allowing them to modify root and shoot growth that resulted in increased survival. Under the drought conditions in this study, carbon transfer did not co-occur with water transfer, indicating plasticity in plant behavioural responses to limiting resources.

Thus far, we have presented only one-sided behavioural responses of plants to resource transfers in MNs (i.e. responses of plants that are recipients of resource transfers or stress signals). This is partly an outcome of study objectives, where experiments were designed to test receiver plant behaviour responses, but may also reflect the importance of relative source and sink strengths within an MN. In a study of AMF networks, for example, Walder *et al.* (2012) demonstrated that flax donated relatively little carbon while receiving 94 % of its nitrogen and phosphorus from the MN, whereas sorghum invested significant amounts of carbon while gaining little, translating into no positive growth effects for sorghum. Interestingly, however, the two plants grown in mixture were more productive relative to when they were grown in monocultures, suggesting unmeasured benefits resulting from linkage to the MN. Likewise, Douglas-fir grew larger when in mixture with linked ponderosa pine, likely due to modified growth behaviour to gain access to excess phosphorus via the MN that would otherwise have been consumed by ponderosa pine as ‘luxury consumption’ (Perry *et al.* 1989). Asymmetrical benefits have also been evident in interspecific carbon transfer from paper birch to Douglas-fir in the summer, with increasing net transfer with shading of Douglas-fir (Simard *et al.* 1997*a*). Regardless of the net effects of carbon transfer through the MN, both Douglas-fir and paper birch benefitted from growing in mixture versus monoculture through increased productivity (Sachs 1996), and from increased resistance to *Armillaria* root disease (Baleshta *et al.* 2005), possibly due to a greater population size of the pathogen-antagonistic rhizosphere microbe, *Pseudomonas fluorescens* (DeLong *et al.* 2002). These studies show that plant behavioural responses to resource transfer via MNs are dynamic, asymmetrical at times and dependant on the identity of the plants, the source–sink patterns in the MN and the environmental conditions of the system (Hynson *et al.* 2012).

Defence signals travelling through the MN clearly result in rapid behavioural responses of recipient plants, and this is evident in sudden changes in foliar defence chemistry (Babikova *et al.* 2013; Song *et al*. 2015) and pest resistance (Song *et al.* 2010, 2014) (see above). For instance, broad beans (*Vicia faba*) responded to aphid attack by swiftly transferring defence signals via the MN to neighbouring bean plants, which responded in turn by producing aphid-repellent chemicals and aphid-predator attractants (Babikova *et al.* 2013). These higher order interactions represent trophic cascades generated by pest infestation triggering signal propagation through the MN. In a different study, defoliation of Douglas-fir resulted in simultaneous transfer of defence signals and carbon to neighbouring healthy ponderosa pine through MNs, resulting in increased defence enzyme production by ponderosa pine, possibly orchestrated by the networking fungus as a strategy to protect itself against the loss of healthy hosts (Song *et al*. 2015). Responses to pest infestations can also lead to larger-scale generational changes in the behaviour of plant-symbiont systems. Shifts in ectomycorrhizal community composition caused by a variety of factors, such as host mortality (e.g. pine beetle outbreaks; Kurz *et al.* 2008), can result in legacy effects that impact future generations of the host species (Karst *et al.* 2015). For example, in areas of western North America dramatically impacted by the mountain pine beetle-induced dieback of lodgepole pine (*Pinus contorta*), EMF have declined significantly (Treu *et al.* 2014). Seedlings grown in soils from beetle-attacked pine stands expressed both reduced biomass and reduced production of monoterpenes compared with those grown in soil from undisturbed pine stands, revealing a transgenerational cascade mediated by fungal symbionts (Karst *et al*. 2015).

In addition to nutrients and defence chemicals, allelochemicals have also been shown to travel between plants through hyphal mycelia (Barto *et al.* 2011; Achatz *et al.* 2014), revealing that not all chemicals moving through the MN benefit the receiving plant. Barto *et al.* (2011) demonstrated that MNs facilitated the transport of natural allelochemicals, thiophenes, as well as the herbicide, imazamox, resulting in decreased growth in receiver plants. Likewise, tomato plants received more jugalone, an allelochemical, and responded with reduced growth when connected to an MN when compared with control treatments lacking an MN (Achatz *et al.* 2014). These examples of amensalism demonstrate that MNs can serve as couriers for biochemical warfare, or negative feedback, from one plant species to another. In previous studies, allelochemicals, demonstrated under laboratory conditions to reduce vigour and growth of recipient plants, were discounted as having meaningful impact *in situ* due to the assumption that they were transmitted through aerial release (Duke 2010). In this manner, they would dilute quickly, precluding a targeted attack. The targeted delivery through an MN, however, demonstrates the important role of MNs in isolating plant behavioural responses to allelochemicals. Thus, the ability of the MN to facilitate allelochemical delivery represents a higher order interaction with direct impacts on the behaviour of the sender and receiver plants.

## Mechanisms for Adaptive Behaviour

Plant genotypes have shown heritability for mycorrhizal fungal traits (Rosado *et al.* 1994*a*; Temel *et al*. 2005) and vice versa (Rosado *et al.* 1994*b*), indicating a mechanism by which MNs play a role in host fitness and adaptive behaviour. At the most basic level, the type of association, whether AMF or EMF, depends on the identity of the plant. Further, within those categories, some plants will associate with a subset of fungal species (Brundrett 2009). The spectrum of symbiotic relationships can range from mutualistic, where both partners benefit, or commensal, where one partner benefits while the other is unaffected, to parasitic where one partner exploits the other (Egger and Hibbett 2004). These are expressed in plant adaptive behaviours such as survival, longevity, growth, physiology, carbon allocation or reproduction. A fungus can express a mutualism with one plant, while simultaneously exploiting a different plant. Mycoheterotrophic plants are perhaps the most extreme example of this type of exploitation, where a plant acquires all of its carbon by parasitizing fungi through the MN (e.g. Leake 1994; Massicotte *et al.* 2012). These plants link into the MN of a nearby tree and siphon off photosynthate, enabling them to survive and grow. Importantly this reveals the existence of a mechanism by which plants can acquire nutritional levels of carbon from mycorrhizal fungi. The fitness of all participants in this scenario is increased by the existence of the MN: (i) the mycorrhizal fungus acquires carbon from the tree (or multiple trees) and may use the mycoheterotroph as the staging ground for long-distance exploration and colonization, (ii) the mycoheterotroph acquires carbon from the fungus and (iii) the tree gains access to a wider pool of soil resources, and potentially connection to other trees facilitating the detection of defence signals.

The relatedness of neighbours in mono-specific plant communities can also influence whether MNs will elicit adaptive behavioural changes. For example, foliar nutrition in AMF *Ambrosia artisifolia* L. improved when it was integrated into an MN with related plants but not conspecific strangers (File *et al.* 2012). Likewise, in mono-specific pairs of EMF interior Douglas-fir grown in greenhouse conditions, foliar micronutrients were increased in kin compared with strangers grown with older conspecifics (Asay 2013). This appears to be linked with mycorrhizal association of this system as mycorrhizal colonization was also elevated in kin seedlings (File *et al.* 2012; Asay 2013). These findings reveal that MNs can play an integral role in kin selection, but the exact mechanisms by which they do this are unclear. However, there is strong evidence that biochemical signals derived from mycorrhizas or roots are involved. For example, Semchenko *et al.* (2014) showed that root exudates carried specific information about the genetic relatedness, population origin and species identity of neighbours, and locally applied exudates triggered different root behaviour responses of neighbours. This included increased root density, achieved through changes in morphology rather than biomass allocation, suggesting the plants limited the energetic cost of their behaviour.

Because the overwhelming majority of plants are predominantly mycorrhizal *in situ*, any root exudates involved in kin recognition are likely to be filtered through mycorrhizal fungi. In a recent study using stable-isotope probing, we found that MNs transmitted more carbon from older ‘donor’ Douglas-fir seedlings to the roots of younger kin ‘receiver’ seedlings than to stranger ‘receiver’ seedlings, suggesting a fitness advantage to genetically related neighbours (Pickles *et al*. unpubl.). This may have been facilitated by the greater mycorrhizal colonization of kin than stranger seedlings (Asay 2013), creating a stronger sink in the MN, an effect also noted in the study by File *et al.* (2012). The greater colonization of kin seedlings may have arisen from complimentary genetics of the fungal genet and tree genotype (e.g. Rosado *et al.* 1994*a*, *b*). In order to establish a mutualism, fungi must overcome a tree's pathogen defence system, which involves the activation of genes to express specific enzymes. Recent work has shown that the EMF species *Laccaria bicolor* secretes an effector protein (MiSSP7) that suppresses host jasmonic acid production, a typical host defence response that interferes with fungal infection, with the effect of enhancing *L. bicolor* colonization of host roots (Plett *et al.* 2014). Furthermore, *Populus* host plants have been shown to induce the production of MiSSP7 in *L. bicolor* through flavonoids present in their root exudates (Plett and Martin 2012). Taken together these findings reveal one mechanism by which plants actively communicate with fungal symbionts to encourage root colonization, with direct consequences for plant behaviour. Thus, related plants may more easily establish mutualisms as a result of the priming effects of root exudates (e.g., Semchenko *et al.* 2014), which may also lower the resource costs of enzyme production, or promote genotypic complementarity between hosts and co-adapted fungal associates. More research is needed to clarify the role of each player in this system. Nonetheless, kin selection is occurring as evidenced by kin versus stranger behaviour differences, and is connected to the formation of MNs, although the mechanism through which the MN elicits the behaviour response remains to be resolved.

## Evolution of MN-Mediated Plant Behaviour

The evolutionary ecology of MNs and the consequences for plant and fungal fitness has been discussed both in terms of individual selection and multi-level or group selection (Perry 1995; Wilkinson 1998; Babikova *et al.* 2013; Johnson and Gilbert 2015). In this section, we revisit these discussions with evidence from our own research investigating the MN effects on plant behaviour. The questions, rephrased from Perry (1995), are: Why should a plant support a mycorrhizal fungus that provides carbon (or nutrients or defence signals) to a competing plant? Why would a fungus pass carbon it acquires from one plant to another plant? Upon reviewing the evidence, we agree with Wilkinson (1998) that individual selection appears sufficient to explain evolution within MNs, but we also concur with Perry (1995) and Whitham *et al.* (2006) that this individual selection can occur within a community context. Several explanations for this are possible, and it is reasonable that a plurality of mechanisms occurs, but the relative contribution of one over the other may vary depending on the conditions of the MN.

Assuming that selection occurs at the individual or gene level, we suggest two possible reasons why a plant would support a fungus that then passes its carbon to another plant. First, if seed dispersal from the parent is limited, as we know is the case in forests (Perry *et al.* 2008), then there could be a high degree of relatedness between parent trees and neighbouring seedlings. Given that nearby neighbours are more likely to be relatives, a plant may pass carbon to an MN that then passes it to a relative, resulting in kin selection. This is inclusive fitness, where it is beneficial to a plant to ensure the shared genes of a related neighbour are passed on, provided the cost to the parent individual is not too high. The occurrence of kin selection in conifers, and the involvement of mycorrhizal fungi in the mechanism, has been demonstrated in our studies with Douglas-fir. We found greater mycorrhizal colonization, micronutrient levels (Asay 2013) and twice as much carbon transferred from established Douglas-fir to nearby kin than stranger neighbours (B. J. Pickles *et al.* unpubl.). This suggests that Douglas-fir trees connected in a *Rhizopogon* network in mono-specific stands (Beiler *et al.* 2010) may behave much like a clonal plant, shuttling resource across the network to related plants in need, increasing the fitness of the evolving genotype. Congruently, we have also found large amounts of carbon (5 % of total photosynthesis) passed through the MN between *Betula nana* plants that are clonal in the Low Arctic Tundra. This carbon transfer appears to be supporting the expansion of related clones through the tundra during warmer periods of the growing season (Deslippe and Simard 2011). But what if the recipient plants are unrelated—that is, they are neither kin seedlings nor ramets? We have found that carbon, albeit in small amounts, can pass from mature Douglas-fir trees through MNs to unrelated Douglas-fir seedlings (Teste *et al.* 2009). Could the transfer of carbon to unrelated seedlings simply be a cost of being linked into MN, or do the donor trees have any control over where and how much carbon is transferred? Our studies point to the latter. Teste *et al.* (2009, 2010) found that Douglas-fir trees transferred more carbon and nitrogen to neighbouring seedlings where the donor tree was larger or the receiver seedlings had a greater demand. Moreover, we have found that Douglas-fir transferred net amounts of carbon to neighbours of a different species, paper birch, which then passed the net amounts of carbon back to Douglas-fir at a different time of year (Philip 2006) and when it was under increasing shade (Simard *et al.* 1997*b*). These studies demonstrated that the amount of carbon transferred depended on the strength of the sink and the input of the donor (Teste *et al.* 2009, 2010), and thus suggest a degree of control by the donor over the amount of carbon passed to the networking fungi, where the donor may donate excess photosynthate to the MN where it is then shuttled based on strength of the sink (Simard *et al.* 2012). From the plant's perspective, the second reason why it would pass carbon to its networking fungus that then passes it to an unrelated plant individual is that there is an evolutionary advantage to the plant through support of the highly diverse and adaptive mycorrhizal symbionts in the MN. The generalist fungal species in the MN can rapidly evolve to the temporally and spatially diverse environment, providing a mechanism for the longer-lived plants and trees to cope with an uncertainty and variability (Wilkinson 1998).

The second question, why a networking fungus would pass carbon from one plant to another, can also be explained by individual selection. If the networking fungus acquires more carbon from one plant than it requires for its own fitness, it can then supply the excess carbon to other networked plants in need, thus diversifying its carbon portfolio for insurance against potential loss of one of the hosts. This more secure carbon source from multiple plants is important to the fitness of the fungal species in variable environments (Perry *et al.* 1989; Wilkinson 1998). From the fungal species perspective, the relatedness of plants involved in the network should be of no consequence, as long as a long-term carbon source is secured. Heil and Karban (2009) similarly suggest that interplant communication via VOC stress signals may be an unavoidable consequence of individual defence strategies or are an extension of within-plant signalling. Interestingly, the greater stability found in mixed species plant communities than monocultures (Tilman *et al.* 2001; Perry *et al.* 2008) suggests that networking and communicating with multiple plant species is a lower risk strategy for the fungus in the MN than networking with only a single plant species. In support of this, we found that ponderosa pine received both carbon and defence signals from damaged neighbouring Douglas-fir through networking mycorrhizal fungi (Song *et al*. 2015). Here, the networking fungus may have acted to protect its net carbon source, by allocating carbon and signals to the healthy, more reliable ponderosa pine. In unstable environments, such as ecosystems under stress and experiencing species turnover as a result of climate change, the MN may therefore benefit from transferring carbon and defence signals interspecifically, favouring hosts that can supply more carbon (Kiers *et al.* 2011). Babikova *et al.* (2013) also suggested that allocating stress signals to plants that supply more carbon would benefit the fungus most. It is possible, therefore, that MN-based transfers and signals have evolved through costs and benefits to favour reciprocation, but also remain more generic among multiple plants species in stressful environments for long-term stability. Nothing is known about the specificity of transfer or signalling pathways through MNs (Johnson and Gilbert 2015), but the differences in specificity of herbivores, and the differences in stability in mixed versus monoculture plant communities, provide fertile grounds for further research.

Group selection is considered to occur where natural selection expresses at the level of the group instead of the individual. It is thought possible where there is an organization of individuals into groups that have a complex social structure that promotes the fitness of group members. When considering that group selection occurs at the level of the species for the formation of groups, it is important to note that there is congruence between individual and group selection theory. Perry (1995) argues that evidence for group selection exists in cooperative guilds, where multiple plants are linked together by MNs for mutual aid. The guilds are then stabilized either through tit-for-tat relationships or reciprocal altruism between the plants and the fungi. It is through these internal relationships and positive feedbacks that the self-organizing behaviour of the guild or group develops. Cooperative guilds exist in communities, especially forests, where species share an ecological commons and experience stochastic disturbance that leads to hiatus in one or more plant species (Perry 1995). There is evidence for both tit-for-tat and reciprocal altruism in MNs in forests, both which would be resistant to cheaters (i.e. individuals that benefit without reciprocating). Tit-for-tat, distinct from mutualisms, is evident in bidirectional transfer between paper birch and Douglas-fir (Simard *et al.* 1997*a*, *b*; Philip *et al.* 2010) and between unrelated Douglas-fir (Teste *et al.* 2010). This cooperative bidirectional exchange occurs over a period of a few days and appears to be related to the behaviour and possibly fitness of the individuals involved in the network. However, reciprocal altruism, or repeated prisoners dilemma, occurs over longer time periods, and this explanation is more congruent with the highly variable disturbances and hiatus in forests. There is some evidence for reciprocal altruism through the switches in the direction of net carbon transfer between paper birch and Douglas-fir (Philip 2006) or maple and trout lily seedlings (Lerat *et al.* 2002) in response to differential changes in plant phenology over a period of several months. The hyperlinking that occurs in Douglas-fir—*Rhizopogon* networks also illustrates that topology of MNs is designed for such complex reciprocal exchanges between trees. The trees in these and other forests are considered foundational species (Simard 2009), and the traits of foundational trees have been shown to have heritable effects on the associated networking mycorrhizal fungi (Rosado *et al.* 1994*a*, *b*), bird and arthropod communities (Whitham *et al.* 2006), soil microbes (Schweitzer *et al.* 2008), and potentially gene regulation of connected neighbours connected (Song *et al*. 2015) and the biochemistry of subsequent generations of seedlings (Karst *et al*. 2015), suggestive of group selection. As such, group selection may help explain why forest productivity and tree disease resistance in communities of Douglas-fir, paper birch, their inter-linking mycorrhizal fungi and associative soil microbes are enhanced compared with monocultures of Douglas-fir alone (Simard *et al.* 2005). However, studies are needed to test whether groups actually do benefit from MN-driven plant behaviour changes.

## Complex Adaptive Systems

Underground ‘tree talk’ is a foundational process in the complex adaptive nature of forest ecosystems. Since plants form the basis of terrestrial ecosystems, their behavioural interactions, feedbacks and influences are important in generating the emergent properties of ecosystems (Levin 2005). Given the connectivity inherent in the formation of MNs and the impressive array of plant behavioural interactions that can be mediated through them, plant behaviour and MNs are intricately linked. In the interior Douglas-fir forests of British Columbia, seedlings regenerate within the MN of old conspecific trees. The architecture of the MN is scale-free, where hub trees are highly connected relative to other trees in the forest (Beiler *et al.* 2010), and this is characteristic of a complex adaptive system (Simard *et al.* 2013; Beiler *et al.* 2015). The scale of the MN is at least on the order of tens of metres (Beiler *et al.* 2010) and potentially much larger, with a single fungus sometimes spanning hundreds of hectares of forest (Ferguson *et al.* 2003). Recent work on the diversity of plant–fungal connections in forests revealed multiple levels of nestedness in the associations between host plants and fungal symbionts (Toju *et al.* 2014; Beiler *et al.* 2015). Each individual component (plant or fungus) of the ecosystem-wide network will, therefore, have a different potential to influence the behaviour of every other individual based on the extent, diversity and hierarchical level of its connections. As discussed above, the connections created by mycorrhizal fungi are agents for both positive (Song *et al.* 2010) and negative (Achatz *et al.* 2014) feedbacks to complex adaptive plant behaviour, which lead to self-organization of ecosystems (Simard *et al.* 2013; Beiler *et al.* 2015). Resilience is an emergent property of the interactions and feedbacks in scale-free networks (Levin 2005). Targeted loss of hub trees, however, can cross thresholds that destabilize ecosystems. Through the study of MNs, we are beginning to characterize the connections that are important to behaviour of system agents and thus ecosystem stability.

## Future Research

With evidence emerging about the potential levels of connectivity in a forest, as well as the extent of the influence that an MN can have, further work to determine the drivers of the senders and receivers, and the nature of the couriers and messages along the mycorrhizal communication highway is all the more relevant. Roots in soil compete (Cahill *et al.* 2010), yet neighbouring trees share vital resources under stress (Philip 2006). The fungi involved may mediate competition between plants and allow for cooperation (Kiers and van der Heijden 2006). Observing the movement of resources and signalling molecules in field situations will give us a better understanding of how the various components discovered in greenhouse experiments actually manifests to generate the complex ecosystem patterns we observe. Thus the creation of field experiments that examine the scale and diversity of plant communication and behavioural responses taking place exclusively through MN are crucial. Further elucidation of the array of plant signalling molecules that can be transported via the MN will help to establish the full suite of belowground communications that are possible. Finally, experiments are needed to better understand selective mechanisms involved in the evolutionary ecology of MNs.

## Sources of Funding

This work is supported by an NSERC CGS-D to M.A.G. and an NSERC Discovery Grant and NSERC CREATE Grant to S.W.S.

## Contributions by the Authors

S.W.S. conceived the idea behind the review, M.A.G. and S.W.S. wrote the first draft, and S.W.S., M.A.G., A.K.A. and B.J.P. contributed to subsequent drafts and provided comments.

## Conflict of Interest Statement

None declared.
